# Extracellular Vesicles and Their Applications in Tumor Diagnostics and Immunotherapy

**DOI:** 10.3390/cells13232031

**Published:** 2024-12-09

**Authors:** Scott Strum, Valentina Evdokimova, Laszlo Radvanyi, Anna Spreafico

**Affiliations:** 1 Princess Margaret Cancer Centre, University Health Network, Toronto, ON M5G 2M9, Canada; 2 Division of Medical Oncology and Hematology, Department of Medicine, University of Toronto, Toronto, ON M5S 3H2, Canada; 3 Ontario Institute for Cancer Research, Toronto, ON M5G 0A3, Canada; 4 Department of Immunology, University of Toronto, Toronto, ON M5S 1A8, Canada

**Keywords:** extracellular vesicles, immunotherapy, clinical trials

## Abstract

Extracellular vesicles (EVs) are cell-derived nanoparticles that have attracted significant attention in the investigation of human health and disease, including cancer biology and its clinical management. Concerning cancer, EVs have been shown to influence numerous aspects of oncogenesis, including tumor proliferation and metastasis. EVs can augment the immune system and have been implicated in virtually all aspects of innate and adaptive immunity. With immunotherapy changing the landscape of cancer treatment across multiple disease sites, it is paramount to understand their mechanisms of action and to further improve upon their efficacy. Despite a rapidly growing body of evidence supporting of the utility of EVs in cancer diagnostics and therapeutics, their application in clinical trials involving solid tumors and immunotherapy remains limited. To date, relatively few trials are known to incorporate EVs in this context, mainly employing them as biomarkers. To help address this gap, this review summarizes known applications of EVs in clinical trials and provides a brief overview of the roles that EVs play in cancer biology, immunology, and their proposed implications in immunotherapy. The impetus to leverage EVs in future clinical trials and correlative studies is crucial, as they are ideally positioned to synergize with advancements in multi-omics research to further therapeutic discovery and our understanding of cancer biology.

## 1. Introduction

One of the most important advancements in the management of solid tumor malignancies over the past decade has been the successful implementation of immune therapies. Immune-based treatments have led to improvements in patient outcomes in numerous cancers, including melanoma [1], non-small cell lung cancer [2], hepatocellular carcinoma [3], renal cell carcinoma [4], and others [5,6,7,8,9]. However, only a minority of all solid tumor patients achieve significant clinical benefit [10], and the mechanisms driving primary and acquired resistance remain under investigation [11,12]. In addition, existing tissue-based immunotherapy biomarkers such as PD-L1 expression, tumor mutational burden (TMB), and microsatellite instability (MSI) have intrinsic limitations, including the invasiveness of sample collection, intra-tumor heterogeneity, and the lack of validation for clinical use in multiple tumor types [13,14,15]. Therefore, in addition to the need for improved immunotherapies, there remains an urgent need for developing biomarkers to optimize immunotherapy management and advance our understanding of treatment resistance. One of the very promising new entities that has gained significant interest in disease biology and cancer management over the past decade are extracellular vesicles (EVs).

EVs are secreted by all mammalian cells [16,17] and serve as critical mediators of extracellular communications between cells and tissues by transporting macromolecular cargo, such as RNA, DNA, lipids, metabolites, signaling molecules, major histocompatibility complexes, and other proteins that are enclosed into a lipid bilayer membrane and protected from degradation [18,19,20,21]. EVs have been shown to play important roles in numerous human disease states, including cardiovascular, neurodegenerative, and autoimmune disorders, viral infections, and cancer [16,22,23]. In addition, EVs can be isolated from virtually all bodily fluids, such as plasma, urine, cerebrospinal fluid, and human milk [16], and may therefore be employed as biomarkers for detecting disease-associated changes, using minimally invasive procedures of sample collection and analysis.

The number of EVs released and their content varies depending on the cell of origin and pathophysiological state of a given cell or tissue. Most importantly, EVs are released by living cells and reflect their metabolic, proliferative, and diseased conditions [23]; this is in contrast to circulating tumor DNA (ctDNA), which is mainly released by dying cells and accumulates during disease progression. In the context of cancer, tumor-derived EVs are instrumental for reprogramming immune and non-immune cells in the tumor microenvironment (TME) and pre-metastatic niche at distant sites by transmitting various RNA species, proteins, lipids, and metabolites systemically and at local tissue sites, many of which are uniquely associated with specific tumor types. As discussed below, cancer-specific cargo may include tumor antigens, immune checkpoint proteins, mutated DNA, mRNAs, microRNAs, and transfer RNAs [20,21]. An emerging field is focused in particular on endogenous retroelements (EREs), including short and long interspersed elements (SINEs and LINEs) and human endogenous retroviruses (HERVs), which are largely silenced in healthy tissues and reactivated during the early stages of cancer development [24,25]. Due to their virus-like features, EREs may play important roles in cancer-associated inflammation through their EV-mediated transfer to immune and non-immune cells in the TME and systemic circulation [25,26,27,28,29].

EVs represent a highly heterogeneous group of nanoparticles, and significant efforts have been made to standardize their isolation and classification [30,31]. The latest classification guidelines categorize them into small EVs (<200 nm) and large EVs (>200 nm) based on their size, which is determined by various biophysical methods, including nanoparticle tracking analysis, electron microscopy, differential centrifugation, and size exclusion chromatography [30,31,32]. The most common purification protocols involve medium-speed centrifugation (10,000–16,000× *g*) to obtain large EVs followed by high-speed centrifugation (100,000× *g*) to collect small EVs. EVs can also be distinguished by their origin from different cellular compartments and by their methods of biogenesis, with two major subtypes: exosomes and ectosomes [33]. Exosomes are small EVs formed by fusion of multi-vesicular late endosomal bodies with the plasma membrane and, as a result, are enriched with proteins involved in endosomal biogenesis and transport [32,33,34]. Among ~1200 core exosomal proteins, syntenin-1 is the most abundant and universal marker for exosomes from different cellular sources [35]. Unlike parental cells, exosomes are enriched with various lipids, including cholesterol, sphingomyelin, ceramide, phosphatidylcholine, and phosphatidylserine, which may facilitate their delivery and internalization by recipient cells [36]. Ectosomes, in contrast, are released directly from plasma membrane and are subdivided into classical vesicles (150–1000 nm diameter), which are positive for Annexin A1 and A2 markers, and larger vesicles, including oncosomes (>1 µm diameter) [33], whose release from tumor cells is driven by oncogenic proteins [32]. Despite differences in their biogenesis, exosomes and small classical ectosomes share similar sizes and common protein markers (most notably, tetraspanins CD9, CD63 and CD81), representing a challenge for their separation and functional characterization. Lastly, apoptotic bodies (50–5000 nm diameter) are released by dying cells in the late stages of apoptosis, and thought to regulate immune responses and inflammation within the TME; they can cause immune tolerance in some cases [32]. These distinctions are important for EV-based development and clinical applications, albeit direct cross-comparisons between studies are difficult due to the lack of universal markers for each EV subpopulation and standardized protocols for their isolation [30,37]. Due to these reasons, attention to the specific methods of EV isolation and characterization are crucial to ensure reproducibility and accuracy of interpretation of results, including evaluation of previously published data. 

**Figure 1:**
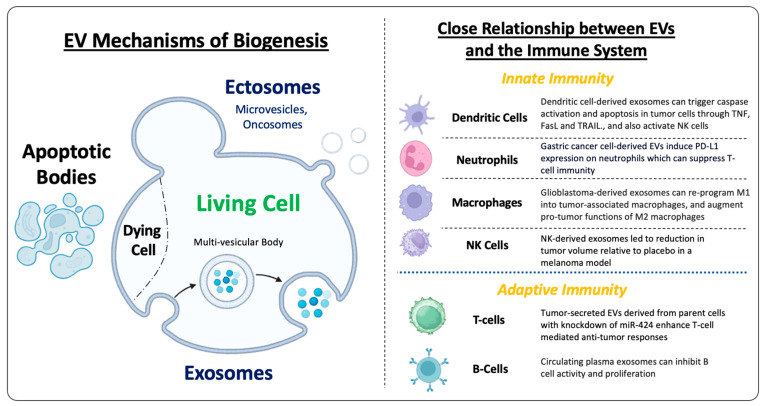
Left: Schematic representation of common mechanisms of EV biogenesis. Exosomes are small EVs (<200 nm diameter) generated through the fusion of multi-vesicular bodies with the plasma membrane. Ectosomes, in contrast, are released directly from the plasma membrane, and are generally subdivided into classical microvesicles (150–1000 nm diameter) and larger vesicles, including oncosomes (> 1 µm diameter). Lastly, apoptotic bodies (50–5000 nm diameter) are released by dying cells in the late stages of apoptosis. Right: Cross-talk between tumor and immune cells EVs. Virtually all innate and adaptive immune cell types are influenced by, or can influence, EVs. A subset of examples from select in vitro or in vivo studies are shown here, including dendritic cells [39], neutrophils [40], macrophages [41], NK cells [42], T-cells [28], and B-cells [43].

EVs have been studied in many biological contexts in human disease, which have been reviewed in detail elsewhere [32,33,34,38]. The main focus of this review is on the role of EVs in solid tumor biology and applications for immunotherapy, including registered clinical trials. EV applications in solid tumor immunotherapy trials remain relatively underexplored, despite strong pre-clinical evidence supporting their use in optimizing treatment selection, enhancing immunotherapy efficacy in some cases, elucidating mechanisms of resistance, and causing immune dysfunction in other cases. As such, we will address the utility of EVs as a source of invaluable non-invasive biomarkers, their unique characteristics, and some specific mechanisms of action, and we will highlight their clinical potential, including the possibility of helping to overcome limitations inherent to conventional immune therapies (Figure 1). 

## 2. EVs in Cancer Biology and Immunoregulation 

EVs play diverse roles in the modulation of the immune system, exhibiting both pro- and anti-tumorigenic effects and regulating virtually all aspects of the innate and adaptive arms of the immune systems [44,45,46]. Their activity has broad implications for improving the understanding of the mechanisms of tumor growth and metastasis, developing treatment approaches, and predicting immunotherapy responsiveness and resistance. For instance, it is broadly accepted that T-cells represent the main anti-tumor effector cell type [47], and they are highly susceptible to modulation by EVs [44,46]. Examples include miR-424-positive EVs from colorectal cancer cells, which are capable of downregulating CD28-CD80/86 costimulatory pathways in T-cells and dendritic cells within the tumor microenvironment, leading to resistance to immune checkpoint blockades in pre-clinical mouse models. Modified tumor-secreted EVs derived from parent cells with the knockdown of miR-424 both enhanced T-cell mediated anti-tumor responses and increased the checkpoint inhibitor response in an orthotopic mouse tumor model [48]. PD-L1, a checkpoint immunosuppressive protein often found on the surface of tumor cells, has also been found in shed tumor EVs, where exosomal PD-L1 was shown to suppress tumor growth and CD8^+^ T-cell activity in a syngeneic mouse melanoma model [49]. Similar findings were reported in a pre-clinical prostate cancer model where PD-L1 knockout in tumor cells depleted a pool of circulating exosomal PD-L1 and suppressed tumor growth associated with enhanced T-cell activation. In contrast, systemically introduced exosomal PD-L1 restored the growth of these tumors by inhibiting anti-tumor immunity [50]. In addition, other studies have found that PD-L1 is detected in plasma exosomes of patients with various types of cancers, including gastric, breast, pancreatic, lung, and esophageal lung cancers and melanoma, indicating that EVs can act as decoys to suppress the anti-tumor activity of cytotoxic T cells [51]. Moreover, exosomal PD-L1 can also mediate anti-PD-L1 antibody resistance by sequestering therapeutic antibodies, which results in the insufficient blockade of tumor PD-L1 [52]. Other tumor EV-driven mechanisms of immunosuppression involve enhanced proliferation and expansion of immunosuppressive Treg cells while impairing proliferation and inducing cytokine release and exhaustion of effector T-cells through the delivery of regulatory miRNAs and enzymatically active arginase-1 (ARG1), as detailed elsewhere [46,53].

Aside from directly modulating adaptive immunity, including B-cells [44], tumor EVs can exert other immunosuppressive effects by creating a chronic inflammatory environment and skewing the differentiation of myeloid cells and stromal fibroblasts towards pro-tumorigenic phenotypes [19,54,55]. Exposure to tumor EVs activates multiple pro-inflammatory pathways in target cells, both locally and systemically. Among the most affected entities are myeloid cells, which have a high capacity to clear different pathogens, cell debris, and EVs from circulation and the local environment. Upon engulfing tumor EVs, circulating monocytes and tissue macrophages can acquire immunosuppressive phenotypes, including myeloid derived suppressor cells (MDSCs) and an M2 macrophage phenotype associated with subdued anti-tumor responses and systemic adaptive immunity [56,57]. In addition, EVs released by these alternatively differentiated myeloid cells were shown to hyperactivate CD8^+^ T-cells, resulting in their exhaustion and in activation-induced cell death [58]. In a syngeneic breast cancer mouse model in which paired MDSC and MDSC-derived exosomes underwent shotgun proteomics and next-generation sequencing, several cargos were differentially expressed, including TGF-β1, which was higher in MDSC EVs relative to parental cells, and has been classically implicated in facilitating immunosuppression [59]. Another important cell type within the TME that is influenced by EV-mediated crosstalk between tumor and stromal environment are cancer-associated fibroblasts (CAFs). CAFs are a heterogenous group of cells having a multitude of functions, some of which include inducing immune cell dysfunction and enhancement of immunotherapy resistance [60]. Melanoma cell-secreted exosomes have been shown to reprogram fibroblasts into proangiogenic CAFs by suppressing SOCS1 expression and activating the JAK/STAT pathway through miR-155 [61]. Ultimately, the EV-mediated interplay between cancer cells, stromal cells, and circulating immune cells and their collective action may predetermine disease stability or cancer progression. 

Beyond pro-tumorigenic effects exerted through modulation of the immune system, EVs have also been implicated in other aspects of cancer development, progression, and metastasis, further underscoring their biological and clinical significance. For example, EVs purified from the plasma of 11 breast cancer patients and 8 healthy individual controls were injected orthotopically into the mammary fat pad of nu/nu mice in combination with MCF10A normal breast cells. Five mice injected with exosomes from breast cancer patients developed tumors, while exosomes from healthy donors had no effect [62]. The proposed mechanism involves processing of precursor miRNAs in tumor EVs, allowing for an efficient and rapid silencing of mRNAs and transcriptomic reprogramming in target nontumorigenic epithelial cells, underscoring the tumorigenic potential of patient-derived serum EVs. A recent study demonstrated the role of EVs containing SINE/Alu RNAs in promoting invasion and metastasis in colorectal cancer patients via induction of epithelial-to-mesenchymal transition (EMT) and NLRP3 inflammasome activation [63]. Among the mechanisms of tumor EV-mediated tumor progression and metastasis are tumor-draining lymph node conditioning via the activation of proangiogenic signaling programs and tissue remodeling [64] and the delivery of various oncogenic mRNAs and proteins that can facilitate the establishment of a pre-metastatic niche [65,66]. A diverse multitude of EV-driven pro-tumorigenic activities not only underscores their critical roles in carcinogenic progression and immunosuppression but also supports their utility for optimizing existing and future immunotherapy protocols for treating solid tumor malignancies through their use as biomarkers of resistance and for their therapeutic targeting to relieve their immunosuppressive effects.

Overall, the number of registered clinical trials employing EVs in the solid tumor immunotherapy space is still limited, representing an unmet clinical opportunity for the development of improved predictive biomarkers and vehicles for immunotherapy. 

## 3. Applications for EVs as Biomarkers and Therapeutic Vehicles in Solid Tumor Immunotherapy 

**Figure 2:**
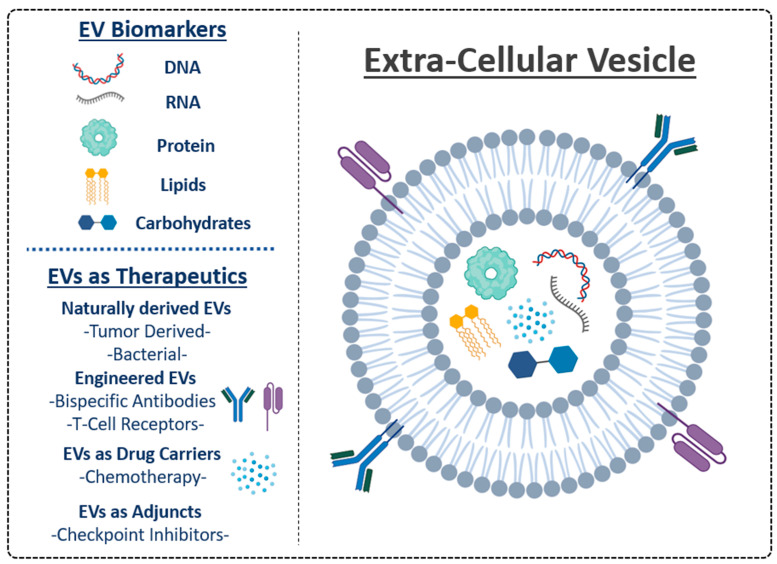
Overview of EV cargo, including DNA, proteins, lipids, and other metabolites, which can be used as non-invasive oncological biomarkers. Several examples are outlined in this review. EVs are also promising therapeutic agents, with mechanisms spanning multiple platforms. To date, EVs have been heavily under-incorporated in immunotherapy-based clinical trials and are positioned to be excellent sources for future exploration in oncology.

Extracellular vesicles (EVs) have been explored for the treatment of various human conditions and diseases, including cancer as well as neurodegenerative, cardiovascular, kidney, and lung diseases [16,22,23,34,37,38]. They hold a series of advantages in clinical application such as biological compatibility, blood–brain barrier penetration, and diversity in the breadth of materials they can carry. For the purposes of clinical application, especially as it relates to oncology, EVs can be broadly utilized in two different ways. The first is though leveraging their natural biological properties to target tissues for treatment purposes, either as a therapeutic agent itself, or as a delivery vehicle for active substances such as cytotoxic chemotherapy. The second way is to use EVs as a biomarker at numerous time points in the course of disease, including diagnosis, therapeutic prediction, treatment optimization, and cancer surveillance (Figure 2). The sections below will elaborate on the most promising EV applications and their potential to advance cancer immunotherapy, with strong supporting pre-clinical evidence to further its exploration in the oncological space. 

### 3.1. EVs as Biomarkers in Solid Tumor Malignancies 

There has been rapidly growing interest in the application of non-invasive biomarkers in the management of solid tumor malignancies, including the investigation of EVs in pre-clinical studies. While blood ctDNA and CTCs have garnered much interest in this space, EVs have numerous unique advantages over CTCs and ctDNA, holding great promise [67]. Compared to CTCs, which are generally very rare (<10 cells per 1 mL of blood) [68,69], EVs can be present in blood plasma at large quantities (over 10^7^ per mL) [70,71]. While only a small proportion of EVs are tumor-derived, EVs are typically increased in the context of cancer [72,73]. Plasma EVs mostly originate from platelets and immune cells, which are expected to be “educated” by the tumor and have the potential to influence both oncogenesis [62,64,65,66,74,75] and the immune system [44]. Compared to ctDNA, EVs have the advantage of holding a multitude of macromolecular analytes available for direct monitoring, including nucleic acids, proteins, and lipids, and can be considered to represent the full landscape of tumor burden in a patient [76]. In contrast, ctDNA arises mainly from apoptotic or necrotic cells and thus is not necessarily reflective of the functional tumor status; it does not represent the entire living tumor cell populations and is usually highly fragmented at the time of measurement [77]. Another important feature of EVs is that they can cross the blood–brain barrier, which has many clinical ramifications [78]. These features collectively position EVs as very promising cancer-associated biomarkers. 

Indeed, EVs have demonstrated positive prognostic and predictive value across multiple cancer types [79,80,81,82,83]. Amongst the most studied to date are levels of PD-L1 and PD-1 in EVs, which have been associated with poor responsiveness to immune checkpoint inhibitor (ICI) therapy in advanced melanoma patients (*n* = 93), with higher PD-1 being specifically associated with poorer progression free survival (PFS) and overall survival (OS) [79]. In another study involving advanced/metastatic non-small cell lung cancer (NSCLC) patients undergoing treatment with chemotherapy with or without ICI therapy (*n* = 72), analyses of pre-treatment levels of PD-L1 in EVs and after 9 weeks of treatment revealed a PD-L1 increase in ICI-treated non-responders relative to that in responders [80]. These authors also demonstrated that the change in PD-L1 EV levels could be an independent predictive marker for both PFS and OS in patients receiving ICIs. In contrast, the associated tumor tissue PD-L1 levels were not predictive of PFS or OS in this cohort of patients. Cytokines that are present in EVs can also be used as cancer-associated biomarkers. Among the most studied is TGF-β, which is classically affiliated with immunosuppressive functions [84,85]. Analyses of TGF-β EV levels in advanced NSCLC patients before and during immunotherapy (*n* = 33) revealed that high baseline TGF-β levels were associated with a lack of response to checkpoint inhibitors, shorter PFS and OS [81]. Importantly, PD-L1 TPS expression in matching tumor tissues from the same patients showed no association with treatment responsiveness.

Aside from EV proteins and cytokines, virtually all types of RNA have been identified in EVs as well, including mRNAs, miRNAs, lncRNAs, piwi-interacting RNAs, srpRNA, and circular RNAs; these have also been implicated in oncogenesis [20,86,87]. In patients with gastric cancer, lncRNA ZFAS1 levels were found to be enriched in patients with gastric adenocarcinoma compared to healthy controls. Furthermore, it was demonstrated that ZFAS1 RNA transmitted to gastric cancer cells directly enhanced cell proliferation and migration and was associated with the lymphatic metastasis and TNM stages [88]. In an analysis of a cohort of 112 breast cancer patients and 60 non-cancer controls, 1551 exosomal lncRNAs were found to be upregulated in breast cancer patients; furthermore, the selection of 11 exosomal lncRNAs were able to distinguish breast cancer patients from healthy controls with a striking AUC of 0.96 (95% CI 0.93–0.99) in the training cohort and an AUC of 0.90 (95% CI 0.81–0.98) in the validation cohort [89]. Similar results have been found in pancreatic cancer where plasma EVs were tested as an early-stage cancer detection tool. The study found that a set of EV-derived proteins detected early-stage pancreatic cancer with an AUC of 0.971 (95% CI  =  0.953–0.986) with 93.3% sensitivity (95% CI: 86.9–96.7) at 91.0% specificity (95% CI: 88.3–93.1). The trained classifier was also validated using an independent cohort of 30 stage I and II cases relative to 83 controls, and it achieved a sensitivity of 90.0% and a specificity of 92.8% [90]. In another study, 30 patients with advanced EGFR/ALK wildtype NSCLC received ICI therapy and their plasma EVs were analyzed by whole transcriptome and small RNA sequencing followed by differential gene expression analysis [91]. Three miRNAs (hsa-miRs 320d, 320c, and 320b) were found to be significantly upregulated in patients with progressive disease at baseline compared to those with partial responses and correlated with unfavorable responses to anti-PD-1 treatment. Lastly, analysis of plasma EV RNA also has been shown to monitor changes in transcriptomic subtypes under treatment selection pressure and to identify molecular pathways associated with recurrence [92]. This study also revealed expressed gene fusions and neoepitopes from EV RNAs.

Exciting new possibilities exist within the realm of endogenous retroelements (EREs) as cancer-associated biomarkers. EREs are estimated to comprise more than 50% of the human genome, are epigenetically silenced in most adult healthy tissues after embryonic development, and are re-expressed in tumors [24,25,93]. They have been implicated in the activation of innate immune responses during the acute phase of inflammation [24]; however, their chronic upregulation may cause chronic inflammation and immunosuppression [94,95]. Multiple studies have detected EREs in the plasma EVs of cancer patients [26,29,96]. For instance, a recent study on Ewing sarcoma showed a selective enrichment of ERE RNAs in EVs relative to their respective parental tumor cells [26]. Furthermore, the authors demonstrated a significant increase of ERE RNA species, especially HERV-K, SINE/Alu, and LINEs, in plasma EVs of Ewing sarcoma patients (*n* = 12) compared to age-matched healthy individuals (*n* = 7; 61%–66% vs. ~13% in healthy donors [26]). Moreover, this study found that tumor EVs can be taken up by myeloid cells and fibroblasts inducing a cGAS-STING mediated chronic innate inflammatory response and DNA damage to these cells [26]. ERE RNAs thus represent a burgeoning repository of comparatively under-explored EV cargo that may hold valuable information about ICI responsiveness and resistance and should be explored as targets in biomarker-based studies. 

Apart from blood, EVs can be detected in other bodily fluids and used as biomarkers [97,98]. In a study of 49 patients with bladder cancer and 48 healthy donors as controls, EVs purified from the urine of patients were significantly enriched with EphA2 [99]. Relative to non-bladder cancer patients, the AUC receiver–operator curve for urinary EV EphA2 was 0.79, indicating the promising accuracy of this test. Furthermore, EVs purified from highly EphA2-expressing bladder cancer cells significantly increased the invasiveness of U-BLC1 bladder cancer cells as assessed by Matrigel invasion assay, whereas the knockout of *EphA2* inhibited this invasion-promoting effect. This study not only identified urinary EV-EphA2 as a novel diagnostic biomarker for bladder cancer but also demonstrated its association with tumor biology and oncogenesis. While it did not involve immunotherapy directly, it provides preliminary support for the exploration of urinary EVs in the solid tumor immunotherapy space, alongside several other studies [100,101,102].

Lastly, EVs are a potential source of genomic information that may offer actionable insights into cancer management. In particular, plasma EVs have been shown to carry identifiable levels of mutated genomic DNA [103], including *KRAS* in pancreatic cancer patients [104,105] and colorectal cancer patients [106]. Comparison of common mutation profiles in tumors, plasma EVs and ctDNA confirmed the high sensitivity of plasma EV testing, which allowed for the detection of *BRAF*, *KRAS*, and *EGFR* mutations present in NSCLC patient tumors (*n* = 43) in 95% of plasma EV nucleic acid samples [107]. Analysis of microsatellite instability (MSI) could be a complementary way to reveal the molecular characteristics of tumors. Using next generation sequencing (NGS) in a small study of 6 patients with colorectal cancer, MSI was accurately detected in ctDNA in 5/6 cases relative to benchmarked paired tumor tissues [108]. It is also noteworthy that a large proportion of plasma ctDNA could be localized in EVs; thus, ctDNA and EV DNA profiles may overlap [109]. Again, although not directly related to immunotherapy, these studies demonstrate the high potential sensitivity of clinical EV DNA and RNA testing as clinical biomarkers, which can be comparable or higher than that of a tumor itself or of plasma ctDNA. 

### 3.2. EVs as Therapeutic Agents in Solid Tumor Malignancies 

EVs have demonstrated therapeutic potential for solid tumor immunotherapy, both as treatments themselves and as adjuncts to enhance the efficacy of existing platforms. This can be facilitated through naturally derived EVs, engineered EVs, or EVs as therapeutic vehicles for the delivery of active agents [38]. Naturally derived EVs are isolated from the conditioned medium of living cells without processing or modification. One example of the successful implementation of naturally derived EVs in pre-clinical settings was described by Seo et al, using EVs isolated from the activated CD8^+^ T-cells of healthy donor mice. Splenocytes from healthy mice underwent CD8^+^ purification through anti-CD28 and anti-CD3 monoclonal antibody (mAb) based selection. Purified CD8^+^ cells were stimulated with recombinant IL-2, and supernatants were used to isolate small EVs using differential ultracentrifugation. The isolated EV pellets from activated CD8^+^ T-cells were injected intra-tumorally into lung metastasis xenograft mouse models and induced an attenuation of tumor progression through a direct EV cytotoxic effect and EV-mediated depletion of mesenchymal tumor stromal cells [110]. 

EVs from activated and antigen-primed dendritic cell derived vesicles (also called “dexosomes”) have been used to positively induce anti-tumor immune responses in pre-clinical and clinical studies [111,112,113,114,115]. One example was demonstrated through the use of an orthotopic mouse model for hepatocellular carcinoma (HCC) [116]. In this study, strong anti-tumor responses were seen when mice treated with dendritic cell exosomes (DEx) that expressed AFP using a lentiviral expression system relative to DEx controls; survival was significantly prolonged as well. Furthermore, this effect was shown to be dependent on an intact immune system, whereby immunodeficient BALB/c nude mice showed minimally reduced tumor retardation relative to immunocompetent mice, suggesting a critical relationship between DEx and T-cell activation against tumor cells [116]. DExes are an exciting platform for further exploration, with numerous advantages over their parent dendritic cells, including relative stability, defined composition, enrichment of peptide-MHC II complexes, and potentially greater dispersibility through tissues and the blood–brain barrier owing to their smaller size [117,118].

Engineered EVs are under development as well, with several features that may help to overcome some of the inherent limitations of naturally derived EVs, such as improvements in tumor targeting, enhanced stability, reduced EV heterogeneity, and enhancements in safety and specificity [54,119]. Modifications in engineered EVs may improve tumor control. For example, genetically engineered exosomes displaying both anti-CD3 and anti-HER2 antibodies were able to dually target T-cell CD3 and breast cancer-associated HER2 receptors as a so-called T-cell engager to activate T cells against HER2 expressing tumor cells. This process was performed through the cloning of bispecific antibody constructs into an endotoxin-free plasmid encoding anti-HER2 and anti-CD3 light and heavy variable regions. The constructs were transfected into Expi293 cells and EVs collected by sequential centrifugation from the culture supernatant. Pellets were re-suspended and characterized by nanoparticle tracking analysis and transmission electron microscopy. Modified exosomes were injected intravenously into xenografted NSG mice bearing HER2-positive human breast cancer cell line HCC 1954, resulting in the significant slowing of tumor growth [120]. No liver or kidney damage or weight changes were observed in these mice. This concept is analogous to bispecific antibodies, which is also based on co-localizing T-cells with tumor cells. It demonstrates the pre-clinical feasibility, efficacy, and safety of engineered exosomes. 

Applications utilizing EVs as drug carriers are also under development. Not only do EVs have the capacity to permeate the blood–brain barrier for enhanced therapeutic efficacy, but they can also improve the pharmacokinetic and pharmacodynamic properties of the encapsulated drug, enhancing efficacy and minimizing toxicity compared to natural delivery systems [121,122]. For example, EGFR-targeting exosomes loaded with anti-tumor miRNA let-7a caused significant tumor regression in an EGFR-expressing xenograft breast cancer mouse model, supporting the therapeutic potential of this approach [123]. Engineered EVs can also be loaded with chemotherapeutic drugs or cytotoxic proteins, and they may also hold great potential for disease control, given their reasonable safety profiles and efficacy [124,125,126]. Methodologically, loading EVs with chemotherapeutic agents is often achieved through the isolation of small EVs by differential centrifugation; this process is followed by the co-incubation of EVs with a cytotoxic agent and either incubation or sonication to assist with encapsulation. Altogether, EVs are readily positioned for further exploration in clinical trials, including the immunotherapy fields. 

EVs may also be useful adjuncts to existing immuno-therapeutics, including ICI therapy. Despite the profound success of ICIs, a significant percentage of patients treated with these agents develop treatment resistance [10,11]. As outlined above, EVs are uniquely positioned to enhance ICI efficacy. One of the obvious targets is PD-L1-positive EVs that counteract anti-PD-L1 therapy by sequestering targeting antibodies and enhancing their clearance by macrophages [52]. PD-L1 was also shown to suppress anti-tumor immune responses and facilitate tumor growth [50]. It is thus expected that blocking PD-L1 expression or release in EVs may enhance ICI efficacy or even sensitize patients with primary resistance. This was demonstrated by using a syngeneic model of prostate cancer with CRISPR/Cas9-mediated deletion of *Rab27a* and *PD-L1* to reduce EV release and block PD-L1 protein expression, respectively. All mice injected with the control prostate cancer cells developed visible tumors within 35 days, whereas mice with *Rab27a*-null and *PD-L1*-null cells showed no tumor growth during the same time period and had significantly longer survival periods [50], supporting PD-L1 targeting as a viable therapeutic strategy.

In addition to conventional ICI therapy, chimeric antigen receptor (CAR)-T cell therapy has had a significant impact on the landscape of cancer treatment, especially for hematologic malignancies. However, progress in the management of solid tumors has been much slower. Not only can the solid tumor TME lead to CAR-T cell exhaustion and anergy, but it can also create a physical barrier that is challenging for CAR-T cells and lymphocytes to permeate [127]. One method of potentially overcoming these barriers is through the use of CAR-T cell EVs. A pre-clinical study of CAR-T cell-derived EVs expressing the CAR construct showed dose-dependent tumor growth inhibition in mouse xenograft models developed from HCC827, MDA-MB-231, and SK-BR-3 breast cancer cell lines [128]. Furthermore, CAR-T exosome treatment appears to be less toxic, as shown by the absence of immune reactions or alterations in animal behavior or weight loss after the intraperitoneal administration of CAR-T exosomes. In comparison, mouse intraperitoneal instillation of CAR-T cells in increasing doses led to symptom progression and ultimately the death of all mice, attributable to cytokine release syndrome (CRS)-like symptomatology. Thus, CAR-T-derived exosomes may be a possible alternative therapeutic strategy to overcome some of the intrinsic limitations of conventional CAR-T cells in solid tumor treatment that has a simultaneously lower risk of cytokine release syndrome. 

### 3.3. Clinical Trials Using EVs as Therapeutic Agents and Biomarkers: An Unrealized Opportunity

As of 1 October 2024, we identified ten registered trials in ClinicalTrials.gov that targeted EVs for cancer immunotherapy and were actively recruiting or completed (Table 1). The search was conducted using a concept block search strategy within each of the associated search fields for extracellular vesicles, solid tumors, and immunotherapy. The relative sparsity of recent clinical trials signals an unrealized potential for the use of EVs as predictive biomarkers, therapeutic targets, or immunotherapy drug delivery vehicles.

Of the registered clinical trials that employed EVs specifically for solid tumor immunotherapy studies, nearly all tested them as biomarkers. Examples include the longitudinal monitoring of exosomal miRNAs in patients with advanced lung squamous cell carcinoma receiving chemoimmunotherapy [129] and longitudinal measurements of exosomal PD-L1 and LAG-3 in conjunction with PD-L1 detection in the circulating tumor cells (CTCs) of hepatocellular carcinoma patients receiving immunotherapy [130]. Furthermore, only two of the identified trials have publicly available results. The first study investigated plasma exosomal lncRNA-GC1 as a potential biomarker in patients with unresectable or metastatic gastric adenocarcinoma on immunotherapy [131,132]. In this trial involving 419 patients across training (*n* = 84), validation (*n* = 255), and prospective cohorts (*n* = 80), plasma EV levels of lncRNA-GC1 were found to be significantly lower in responders than in non-responders. Additionally, baseline EV lncRNA-GC1 positively correlated with progression-free survival (PFS) and overall survival (OS). As a dynamic biomarker, EV lncRNA-GC1 levels were statistically lower at complete response/partial response relative to progressive disease/stable disease. Interestingly, EV lncRNA-GC1 and EV PD-L1 did not show any significant differences between paired tissue PD-L1 CPS-positive (>1) and CPS-negative (0) samples. While this study remains as a preprint [132], it does suggest an independent prognostic and predictive value of exosomal analytes in the therapeutic space relative to conventional tumor PD-L1 detection. 

**Figure 3:**
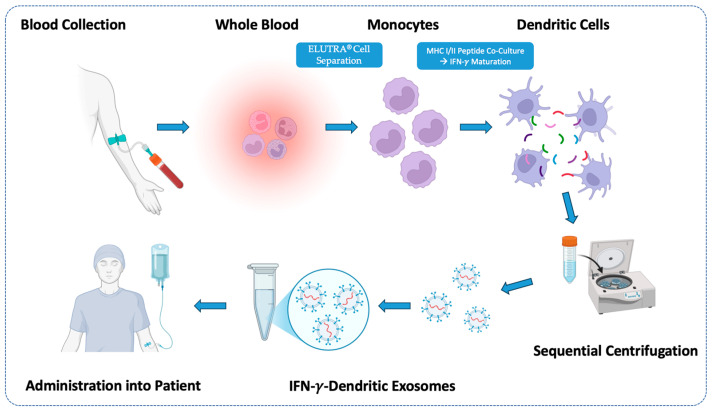
Overview of the methodologic workflow overview of the phase II clinical trial for patients with stage IIIB and IV unresectable NSCLC, using EVs from the MHC I/II peptide-loaded DCs maintenance immunotherapy after induction chemotherapy [134]. Monocytes from patient blood were isolated using ELUTRA® (CaribianBCT, Zaventem, Belgium) and differentiated into dendritic cells using recombinant GM-CSF. Dendritic cells were then loaded with MHC I/II restricted peptides and stimulated to maturation with IFN-*γ*. Dendritic exosomes were then harvested from the conditioned medium of these cells using sequential centrifugation and administered into patients to boost NK and T cell immune responses.

Another promising application that needs more clinical study is the use of tumor cell- or dendritic cell-derived EVs as potential cancer vaccines by priming tumor antigen-specific T-cell responses. The advantage here is that the EVs or exosomes from these sources already express a heterogeneous set of tumor antigens for the priming of tumor-specific responses without the need to painstakingly identify the personal neo-antigen profile of each patient. For example, a phase II trial of 22 patients investigated the benefit of IFN-*γ*-matured dendritic cell exosomes, also called “dexosomes” (IFN-*γ*-Dex), loaded with MHC class I- and class II- restricted cancer antigens as a maintenance immunotherapy after chemotherapy induction in patients with stage IIIB and IV unresectable NSCLC without tumor progression [133,134] (Figure 3). The primary endpoint was to observe at least 50% of patients with PFS four months after chemotherapy cessation. Briefly, monocytes from patients’ blood were isolated using ELUTRA^®^ cell separator (CaribianBCT, Zaventem, Belgium) and differentiated into dendritic cells (DCs) using recombinant GM-CSF. These DCs were then loaded with MHC-I and MHC-II peptides, and DC maturation was stimulated with IFN-*γ*. The IFN-*γ*-Dex were thereafter isolated from the DC conditioned medium using sequential centrifugation and injected into patients as part of the trial protocol. Although the primary endpoint was not met, IFN- *γ*-Dex therapy was well tolerated and its production was feasible. Moreover, increased activity of natural killer (NK) cells was observed, supporting further development of this immunotherapeutic strategy [133,134].

## 4. Limitations of EVs in Practice

Although EVs hold tremendous potential in solid tumor disease management, including their excellent biocompatibility, low immunogenicity, high circulatory stability, and penetration of the blood–brain barrier [135], they have inherent limitations. First and foremost, there remains inconsistency in methods of EV isolation, which can lead to variability in assay results [37]. Isolation methods include ultracentrifugation, size exclusion chromatography, immune affinity isolation, and phase separation using microfluidic polymers. Efforts have been made to standardize isolation and characterization of EVs [30,31], but there is yet to be a universally adopted approach across all studies, including clinical trials. Furthermore, EVs are inherently heterogeneous not only in their size but also in their cargo content [136]. Lastly, EVs can be affected by difficult-to-assess factors, including person’s physical activity and other lifestyle habits [137], as well as by age, gender, and ethnicity [138]. For instance, an exploratory study of exosomal proteomic profiling in males with prostate cancer across Caucasian, African American, and Hispanic ethnicities (9 controls and 4 Caucasian, 4 African American, and 4 Hispanic patients) identified common and ethnic background-specific proteins, which were also distinct from those of age-matched healthy male volunteers [138]. This underscores the importance of awareness of potential biases in study design when incorporating these EVs into clinical trials as biomarkers or therapeutics. It is also important to consider the external validity of study results relative to a real-world population. Notwithstanding these limitations, the apparent benefits strongly favor the further utilization of EVs in cancer management and clinical trials. 

## 5. Conclusions 

EVs are non-invasive, standardizable nanoparticles that hold great promise for developing novel solid tumor immunotherapy strategies, including biomarkers, direct therapeutic agents or the targeting of EV-associated immunosuppressive mechanisms. A significant gap still exists in their incorporation into clinical trials, with only a few registered trials currently evaluating their utility. However, there is a growing body of evidence to support their continued pre-clinical study and targeting in clinical trials. While the search strategy employed here within ClinicalTrials.gov may not have captured all trials that employ EVs, it represents a structured approach and underscores the need for greater transparency around correlative study documentation through publicly available platforms. This is particularly crucial given numerous promising pre-clinical and clinical data supporting the utility of EVs for liquid biopsy biomarkers and solid tumor immunotherapy. The major gaps that remain in the management of patients on immunotherapy are to identify non-responders and those predicted to respond to a particular therapy and experience clinical benefit. 

EV-based companion and complementary diagnostic tests and anti-cancer therapies are positioned to intimately change and enhance patient care. Importantly, when a promising candidate is identified in pre-clinical and clinical spaces, regulatory safeguards and considerations should always be factored in, especially given that classifications remain somewhat heterogeneous [139]. Categorizing EVs and optimizing their manufacturing processes at scale is crucial [139,140], even at the early stages of development. Standardizing EV nomenclature, structure, and properties, with specifications that address the origin of cells, modifications, and impurities (e.g., microbial, viral, and chemical agents) is fundamental to this process [30,141]. Validating manufacturing practices, analytical methods, and quality control procedures is essential to ensure their safety and reproducibility as they are often requisites for regulatory approval. Notwithstanding some of these considerations, there is significant potential in this space for EVs, for which the European Medicines Agency (EMA) has already recognized two EV-based medicinal products [142]. Cancer immunotherapy applications should similarly be pursued for many of the aforementioned reasons. 

Lastly, to maximize the potential of EVs in solid tumor immunotherapy, exploring their utility across all EV subtypes, macromolecular cargos, and optimization methods should be pursued. Alternative approaches may involve EVs from other organisms, including plants and bacteria. For instance, intravenous administration of EVs from genetically modified *E. coli* into mice transplanted with CT26 murine colon adenocarcinoma led to dose-dependent reduction in tumor volume, including complete responses with no notable adverse effects [143]. Moreover, these *E. coli* EVs were primarily detected in tumors and not in healthy tissues, suggesting intrinsic targeting potential. Thus, exploring all avenues for the clinical implementation of naturally occurring and engineered EVs will help to realize their therapeutic potential. 

In conclusion, the inclusion of EV-based biomarkers and the testing of EVs as therapeutic vehicles for immunomodulation and drug delivery for solid tumor immunotherapy represents an unmet need that can open new avenues for effective clinical management; however, it is currently under-represented. EVs have been implicated in virtually all aspects of cancer development, immune evasion, immune priming, and immunosuppression, affecting innate and adaptive branches of the immune system. Given their favorable biological characteristics, their presence in all bodily fluids, their relatively simple and cheap methods of isolation, and the plethora of pre-clinical evidence supporting their utility for cancer detection and immune therapy, EVs are positioned to meaningfully improve patient outcomes, especially for patients who do not benefit from conventional immune therapies. 

## Figures and Tables

**Table 1:** Selection of Registered Clinical Trials Involving Extracellular Vesicles and Immunotherapy.

Registered Clinical Trial	Disease Site	Immunotherapy	EV Subtype	Role of EV (Biomarker, Therapy, etc.)	Trial Type	Trial Status
NCT05854030	NSCLC	Anti-PD-L1	Exosome	Biomarker (Exosome miRNA)	Observational	Recruiting
NCT05705583	RCC	Anti-PD-1/PD-L1	Exosome	Biomarker (Exosome)	Observational	Recruiting
NCT05575622	HCC	Immunotherapy	Exosome	Biomarker (Exosome PD-L1 and LAG-3)	Observational	Recruiting
NCT01159288	NSCLC	Exosome vaccination	Exosome	Therapeutic (antigen-loaded DC-derived exosomes)	Interventional	Completed
NCT05427227	GI	Immunotherapy, anti-HER2, or anti-CLDN18.2	Exosome	Biomarker (Exosome tumor-associated proteins)	Observational	Recruiting
NCT05334849	Gastric	Immunotherapy	Exosome	Biomarker (Exosome LncRNA-GC1)	Observational	Completed
NCT05864534	GBM	Anti-PD-1 and anti-CTLA-4	Exosome	Biomarker (Exosome)	Interventional	Recruiting
NCT03576612	GBM	Immunotherapy	EV	Biomarker (EV)	Interventional	Active, not recruiting
NCT04581382	Melanoma	Anti-PD-1	EV	Biomarker (EV)	Interventional	Active, not recruiting
NCT05744076	Melanoma	Immunotherapy	Exosomes	Biomarker (Exosome PD-L1)	Observational	Active, not recruiting

Legend: NSCLC: non-small cell lung cancer. RCC: renal cell carcinoma. HCC: hepatocellular carcinoma. GI: gastrointestinal malignancies. Gastric: gastric adenocarcinoma. GBM: glioblastoma multiforme. EV: extracellular vesicle.

## Data Availability

No new data were created or analyzed in this study.
